# Associations between Parity, Hair Hormone Profiles during Pregnancy and Lactation, and Infant Development in Rhesus Monkeys (*Macaca mulatta*)

**DOI:** 10.1371/journal.pone.0131692

**Published:** 2015-07-14

**Authors:** Amanda M. Dettmer, Kendra L. Rosenberg, Stephen J. Suomi, Jerrold S. Meyer, Melinda A. Novak

**Affiliations:** 1 Laboratory of Comparative Ethology, Eunice Kennedy Shriver National Institute of Child Health & Human Development, NIH, Poolesville, Maryland, United States of America; 2 Department of Psychological and Brain Sciences, University of Massachusetts Amherst, Amherst, Massachusetts, United States of America; INRA, FRANCE

## Abstract

Studies examining hormones throughout pregnancy and lactation in women have been limited to single, or a few repeated, short-term measures of endocrine activity. Furthermore, potential differences in chronic hormonal changes across pregnancy/lactation between first-time and experienced mothers are not well understood, especially as they relate to infant development. Hormone concentrations in hair provide long-term assessments of hormone production, and studying these measures in non-human primates allows for repeated sampling under controlled conditions that are difficult to achieve in humans. We studied hormonal profiles in the hair of 26 female rhesus monkeys (*Macaca mulatta*, n=12 primiparous), to determine the influences of parity on chronic levels of cortisol (hair cortisol concentration, HCC) and progesterone (hair progesterone concentration, HPC) during early- to mid-pregnancy (PREG1), in late pregnancy/early lactation (PREG2/LACT1), and in peak lactation (LACT2). We also assessed infants’ neurobehavioral development across the first month of life. After controlling for age and stage of pregnancy at the first hair sampling period, we found that HCCs overall peaked in PREG2/LACT1 (p=0.02), but only in primiparous monkeys (p<0.001). HPCs declined across pregnancy and lactation for all monkeys (p<0.01), and primiparous monkeys had higher HPCs overall than multiparous monkeys (p=0.02). Infants of primiparous mothers had lower sensorimotor reflex scores (p=0.02) and tended to be more irritable (p=0.05) and less consolable (p=0.08) in the first month of life. Moreover, across all subjects, HCCs in PREG2/LACT1 were positively correlated with irritability (r_(s)_=0.43, p=0.03) and negatively correlated with sensorimotor scores (r_(s)_=-0.41, p=0.04). Together, the present results indicate that primiparity influences both chronic maternal hormonal profiles and infant development. These effects may, in part, reflect differential reproductive and maternal effort in mothers with varied caretaking experience. In addition, infant exposure to relatively higher levels of maternal cortisol during the late fetal and early postnatal periods is predictive of poorer developmental outcomes.

## Introduction

Pregnancy and lactation are known to affect circulating levels of ovarian steroids (e.g., estradiol and progesterone, among others) and glucocorticoids (e.g., cortisol and corticosterone) in many mammalian taxa including rodents, bovines, nonhuman primates, and humans. Researchers examining fluctuations in these hormones have largely relied on analyses of serum, salivary, or fecal concentrations, and their findings are generally in agreement: estradiol gradually rises throughout pregnancy and peaks just prior to parturition, then sharply declines shortly afterward, whereas progesterone remains elevated throughout pregnancy and sharply declines just prior to parturition [1]. Both hormones remain low during lactation [2, 3]. In contrast, cortisol increases throughout pregnancy, peaks at parturition, and sharply declines shortly after parturition [2]. When comparing lactating and non-lactating animals, however, the picture is murky: some studies have shown that plasma and fecal levels of ovarian steroids and glucocorticoids differ in lactating versus non-lactating animals [4–6] whereas others have shown no difference [7–9]. In rodents and humans, lactation appears to have an attenuating effect on hypothalamic-pituitary-adrenal (HPA) axis responses to stressful stimuli [10, 11].

Parity may also influence circulating levels of these hormones, though here too the results are mixed: primiparous nonhuman primate females exhibited higher fecal estrogen metabolites [2] but lower serum estradiol [12] than multiparous females. In cows, primiparity compared to multiparity was associated with a longer interval from parturition to the first progesterone peak, which is an indicator of likelihood of conception/implantation [13, 14]. With respect to cortisol, studies in women have shown that multiparous mothers exhibit blunted salivary cortisol responses to stress compared to primiparous mothers [15].

Parity is known to influence pregnancy outcomes, birth weight, and infant growth, with less experienced mothers having more complications and smaller/slower growing babies [16–18]. However, parity effects on other measures of infant development, such as neurobehavioral indices, remain largely unexplored. With respect to physiological variables, higher levels of maternal stress and/or cortisol in pregnancy and infancy are known to negatively impact pregnancy outcome [19] and infant emotional and cognitive development [20–22], though some studies have suggested that these outcomes depend on the timing of this exposure [23, 24].

Possible reasons for some of the inconsistencies in these studies include the diversity of species being examined and the matrix for assessing hormone concentrations. The majority of studies investigating pregnancy, parity, and hormone function have been conducted with rodents and cows, which undergo estrous cycles and absorb their endometrium. In contrast, human and nonhuman primates experience monthly menstrual cycles that result in the shedding of the endometrium. These differences may account for many of the inconsistencies across studies. However, another important contributing factor may be the type of sample used to assess hormone concentrations. Different types of biological fluids and tissues yield different information regarding the timing of physiological responsivity, ranging from minutes (as measured in cerebrospinal fluid, plasma, and saliva), to hours (urine), to one or more days (feces, depending on species gut passage time), to weeks or months (hair). The short-term, or “point”, samples (i.e., serum, plasma, or saliva) are significantly affected by environmental factors including time of day, meal consumption, and stressful stimuli. Thus, in order to gain a thorough understanding of the long-term hormonal profiles of pregnant females or mothers differing in parity, one must collect numerous repeated samples over many months or years [25]. In the case of animal studies, continual “point” sampling for monitoring reproductive cycles and pregnancy/lactation either requires an indwelling catheter [26, 27] or repeated restraint and venipuncture, which may affect the resulting sample owing to the inherent lability of the hormones being measured [25]. In socially living animals (captive or wild), obtaining reliable urine or fecal samples to assay hormones over hours or days can be logistically difficult and also requires repeated, disruptive sampling to obtain information over the course of weeks or months [28].

Measuring hormones in hair has become a valuable method over the past several years, as this matrix reflects long-term hormonal secretion over many months [25, 29, 30]. The most commonly studied hormone in hair is cortisol, and early research by our laboratory showed that hair cortisol concentrations (HCCs) reflect long-term HPA axis functioning in macaque monkeys [31, 32]. Subsequent studies by a number of research groups have demonstrated that HCCs are a reliable biomarker for stressful life events in humans and various animal species [30, 33–38]. Reproductive hormones have also been detected in human and animal hair [39–42], and hair progesterone has been validated as a means for detecting early pregnancy in cows [43]. Thus, measuring hormones in hair is a useful means of assessing long-term HPA axis and ovarian steroid functioning.

Despite the advent of hair hormone assays, no studies to date have examined long-term HPA axis or ovarian steroid concentrations in primates throughout the periods of pregnancy and lactation, especially with respect to parity. To our knowledge, only three studies have assessed changes in hair cortisol during pregnancy, two in humans [44, 45] and the third in pig-tailed macaque monkeys [46]. All of these studies found that HCCs increase during the later stages of pregnancy. The study by D’Anna-Hernandez and colleagues [44] extended their assessment to three months postpartum and found that mothers’ HCCs significantly declined from the third trimester to this time point, which is in line with a very recent study in sows that reported higher hair cortisol in pregnancy than at weaning [47]. However, in the human studies, no information was provided as to whether or not the subjects were breastfeeding; thus, there is a gap of knowledge surrounding chronic hormone secretion during lactation in primates. Furthermore, these studies also did not take parity into account.

Additionally, no studies to date have examined how long-term measures of maternal hormone production, such as those measured in hair, influence infant development. Numerous studies have reported links between plasma or salivary concentrations of maternal cortisol in pregnancy and/or lactation and infant neurological development [20–22, 24], but these studies typically rely on samples collected once or only a few times and do not reflect chronic hormone secretion that the fetus may be exposed to. Other maternal hormones, such as reproductive hormones (e.g., estradiol and progesterone), are typically studied with respect to maternal care [48] and not measures of infant neurobehavioral development *per se*.

We sought to fill a significant gap in the existing literature by examining long-term hormonal functioning during pregnancy, early lactation, and late lactation (just prior to the onset of the next breeding season) and subsequent infant development in a medically relevant model of pregnancy in humans, the rhesus monkey (*Macaca mulatta*). Like humans, rhesus monkeys have monthly menstrual cycles, long gestations, and provide intensive care for their infants; they are also more similar to humans genetically, physiologically, and behaviorally than are other typically studied models of reproduction like rodents and livestock [49, 50]. We investigated hair concentrations of cortisol and progesterone during pregnancy and throughout lactation to determine whether these profiles changed over time and differed by parity. We also assessed infant neurobehavioral development throughout the first month of life.

We predicted that changes in hair levels of these hormones would be similar to those found in repeated plasma samples, in that they would be higher in pregnancy than during lactation. We also expected to see differences in primiparous compared to multiparous mothers, especially in hair cortisol, given that primiparous rhesus mothers are naturally younger than multiparous mothers [51] and may experience more physiological stress during the peripartum period due to the need to allocate resources to their own continued growth and development in addition to that of their fetus/infant. Finally, we predicted that higher levels of maternal cortisol would correlate negatively with infant neurobehavioral development. Because the relationship between maternal progesterone and infant development is not well understood, we made no specific predictions in this regard but instead explored all possible associations.

## Materials and Methods

### Subjects

This study was carried out in strict accordance with the recommendations in the Guide for the Care and Use of Laboratory Animals of the National Institutes of Health. All procedures were approved by the NICHD Animal Care and Use Committee (Protocol number 11–043).

Subjects were 26 female rhesus macaques (*Macaca mulatta*), aged 5–19 years (mean±SEM: 8.02±0.5), weighing 6.3–10.8kg (mean±SEM: 8.6±0.3) born and raised at the Laboratory of Comparative Ethology at the NIH Animal Center in Poolesville, MD. Monkeys were studied during the 2013 calendar year and were socially housed in large indoor-outdoor runs in stable social groups containing 6–8 other adult females, 1 adult male, and 3–5 offspring. The indoor-outdoor enclosure was constructed of galvanized steel mesh connected by guillotine doors. The floor was covered with wood chips, and multiple perches, swings, and enrichment devices were provided. The indoor pen measured 2.44m x 3.05m x 2.21m, and the outdoor pen measured 2.44m x 3.0m x 2.44m. Animals were given free access between the indoor and outdoor portions except when confined to one half for cleaning (twice per week), laboratory or protocol procedures, or inclement weather (e.g., 4°C or below, a very rare occurrence). Inside lighting was maintained on a 12:12 cycle (0700–1900), and the outdoor portion of the enclosure was exposed to ambient lighting. Monkeys were fed Purina High Protein Monkey Chow (#5038, St. Louis, MO) twice daily and received water *ad libitum*. Supplemental fruit and other foraging materials such as peanuts or sunflower seeds were provided daily [52, 53].

Monkeys were classified as primiparous (PRIMIP, n = 12) or multiparous (MULTIP, n = 14) based on confirmation of pregnancy via ultrasound during routine health exams from January through March 2013. Subject characteristics are displayed in Table 1.

**Table 1:** Subject characteristics for this study.

		AGE GROUP		
PARITY	PREGNANCY STAGE	5-9yrs	10-20yrs	TOTAL
Primiparous (PRIMIP)	Early Preg	6		6
	Late Preg	6		6
	Total	12		12
Multiparous (MULTIP)	Early Preg	1	3	4
	Late Preg	5	5	10
	Total	6	8	14
TOTAL	Early Preg	7	3	10
	Late Preg	11	5	16
GRAND TOTAL		18	8	26

### Hair sampling

Hair samples were collected by shaving the nape of the neck using commercially available pet grooming clippers. Samples were collected during routine health exams in January, April, July, and October 2013, with the same sampling area shaved at each time point. Consequently, hormone concentrations in these samples reflected chronic steroid deposition over the previous time period (specifically three months in the case of the April, July and October samples) [32]. Rhesus monkey gestation is generally considered to last approximately 165 days [54], though in our colony average gestation is closer to 170 days (unpublished data). By the July health exam, all pregnant monkeys had delivered their infants. The April hair sample represented average hormone concentrations during mid-to-late gestation (PREG1; range: 46–182 gestational days; mean±SEM = 124.9±8.7 gestational days), the July samples represented average hormone concentrations in the perinatal period (that is, in late gestation/early lactation; PREG2/LACT1; range: 144–287 gestational days; mean±SEM = 230.1±8.7 gestational days or 60.1±8.7 postnatal days), and the October samples represented average hormone concentrations during peak/late lactation (LACT2; range: 80–221 postnatal days; mean±SEM = 159.2±8.8 postnatal days). In rhesus monkeys, peak lactation occurs between 3–4 postnatal months and tapers significantly by 6 months, with full weaning usually occurring by one year of age when the next sibling is born [55, 56].

### Hormone assays

#### Hair storage and processing

Hair samples were stored in the freezer (-20 or -80°C) in aluminum foil pouches until processing according to the methods described in Davenport et al. (32). Briefly, 250 ± 1.5 mg of hair from each sample was weighed and then placed into a disposable 15 mL polypropylene centrifuge tube. Hair samples were washed twice with 5 mL of isopropanol to remove external contaminants and then air dried in a fume hood. Once completely dry, each sample was placed into a 10 mL stainless steel grinding jar with a 12 mm stainless steel ball and ground to a fine powder using a Retsch ball (model MM200) mill. 50.0 ± 0.5 mg of powdered hair was weighed and placed into a 2.0 mL Eppendorf microcentrifuge tube for extraction.

#### Cortisol extraction and assay

1.0 mL of methanol was added to the powdered hair and the tubes were placed on a constant slow rotator overnight (18–24 hours). Samples were centrifuged for 1.5 minutes at 14,000 RPM in a microcentrifuge to pellet the powder. 0.6 mL of the methanol extract was placed into a 1.5 mL Eppendorf tube, after which the methanol was evaporated using a Savant Speedvac with refrigerated vapor trap and then the cortisol was reconstituted with 0.4 mL diluent from the assay kit. Extracts were either placed in a freezer or assayed immediately. Extracts were analyzed in duplicate for cortisol by enzyme immunoassay (Salimetrics, State College, PA). The intra-assay coefficient of variation was 1.4% and inter-assay coefficient of variation was 5.7%.

#### Progesterone extraction, purification, and assay

Hair progesterone was obtained from the same methanol extracts as used for cortisol analysis. Initial testing of serially diluted methanol extracts for parallelism with authentic progesterone standards revealed the presence of one or more contaminants that interfered in the enzyme immunoassay. Consequently, the following solid-phase extraction procedure was used to purify the samples prior to analysis. 0.6 mL of the methanol extract was placed into a 13 x 100 mm glass tube and dried down under nitrogen gas at 30°C using a water bath. Evaporated extracts were stored at -20°C until further processing. Extracts were reconstituted in 1.10 mL of 30% methanol in deionized water and mixed thoroughly. 1.0 mL of each extract was then loaded onto an Oasis HLB 1cc (30mg) extraction cartridge (Waters, Milford, MA) that had been conditioned according to manufacturer’s instructions. The cartridges were washed with 1.0 mL of 20% methanol in deionized water, after which the progesterone was eluted with 1.0 mL of pure methanol. The purified extracts were dried down under nitrogen at 30°C, reconstituted in 0.45 mL of diluent from the assay kit, and then either placed in a -20°C freezer or assayed immediately. Extracts were analyzed in duplicate for progesterone by enzyme immunoassay (Salimetrics, State College, PA). Progesterone recovery from the extraction cartridges was determined by spiking four separate hair extracts with known amounts of progesterone and then measuring the levels in the unspiked and spiked samples after the purification step. Three additional purified extracts were subjected to serial dilution for determination of parallelism with authentic progesterone standards provided in the assay kit. The intra-assay coefficient of variation for the progesterone assay was 1.6% and the inter-assay coefficient of variation was 6.5%.

#### Neurobehavioral Assessments

Infants were administered routine neonatal assessments of neurological and behavioral development weekly for the first month of life i.e., [57–59]. These assessments lasted approximately 30 min and evaluated infants’ survival reflexes (rooting, sucking, startle), motor reflexes (grasping, clasping, and placing), and sensorimotor reflexes (auditory and visual orientation, and visual tracking). These measures were coded on a scale ranging from 0 (reflex absent) to 1 (weak reflex) to 2 (full/strong reflex). Behavioral indices of emotionality (irritability and easy of consoling) were also recorded. For these measures, researchers gave each infant a score reflecting their emotional reactivity across the entire assessment, ranging from 0 (extremely irritable and inconsolable) to 1 (slightly irritable on a few measures and difficult to console) to 2 (not irritable and very easy to console).

#### Data analysis

All HCC and HPC values were log-transformed to meet the assumptions of normal distribution prior to analysis, but the untransformed values are presented for clarity. Infant sex did not influence any hair hormone measure; however, preliminary correlational analyses revealed that dams’ age in years was significantly negatively correlated with HCC values in July and with HPC values in October, and that gestational day was significantly negatively correlated with HCC and HPC in July. Consequently, monkeys were divided into pregnancy stages based on the first sample collection in April (1 = first half of pregnancy, n = 10; 2 = second half of pregnancy, n = 15). Then, age and pregnancy groups were entered as covariates in repeated measures ANOVAs with time as the within-subjects variable (PREG1, PREG2/LACT1, and LACT2), parity as the between-subjects variable (PRIMIP and MULTIP), and HCC and HPC as the dependent variables. Post-hoc tests with Fisher’s LSD corrections for multiple comparisons were used to determine differences between parity groups for each hormone at each time point. Brazelton scores were averaged into a mean score for the entire first 30 postnatal days, and were analyzed with independent samples t-tests using parity as the grouping variable. Finally, Spearman correlations were used to examine the relations between hair hormone concentrations and Brazelton scores. IBM SPSS v22 was used for analysis, and p≤0.05 was considered statistically significant.

## Results

Recovery of progesterone from spiked hair extracts following solid-phase extraction averaged 109.7% (n = 4). Parallelism tests of serially diluted hair extracts with authentic progesterone standards yielded an average R^2^ value of 0.99 (n = 3).

Even after controlling for females’ ages and pregnancy stages, while no effects of infant sex were evident, significant effects of parity were revealed for both hair cortisol and hair progesterone.

### Hair cortisol concentrations (HCCs)

**Fig 1:**
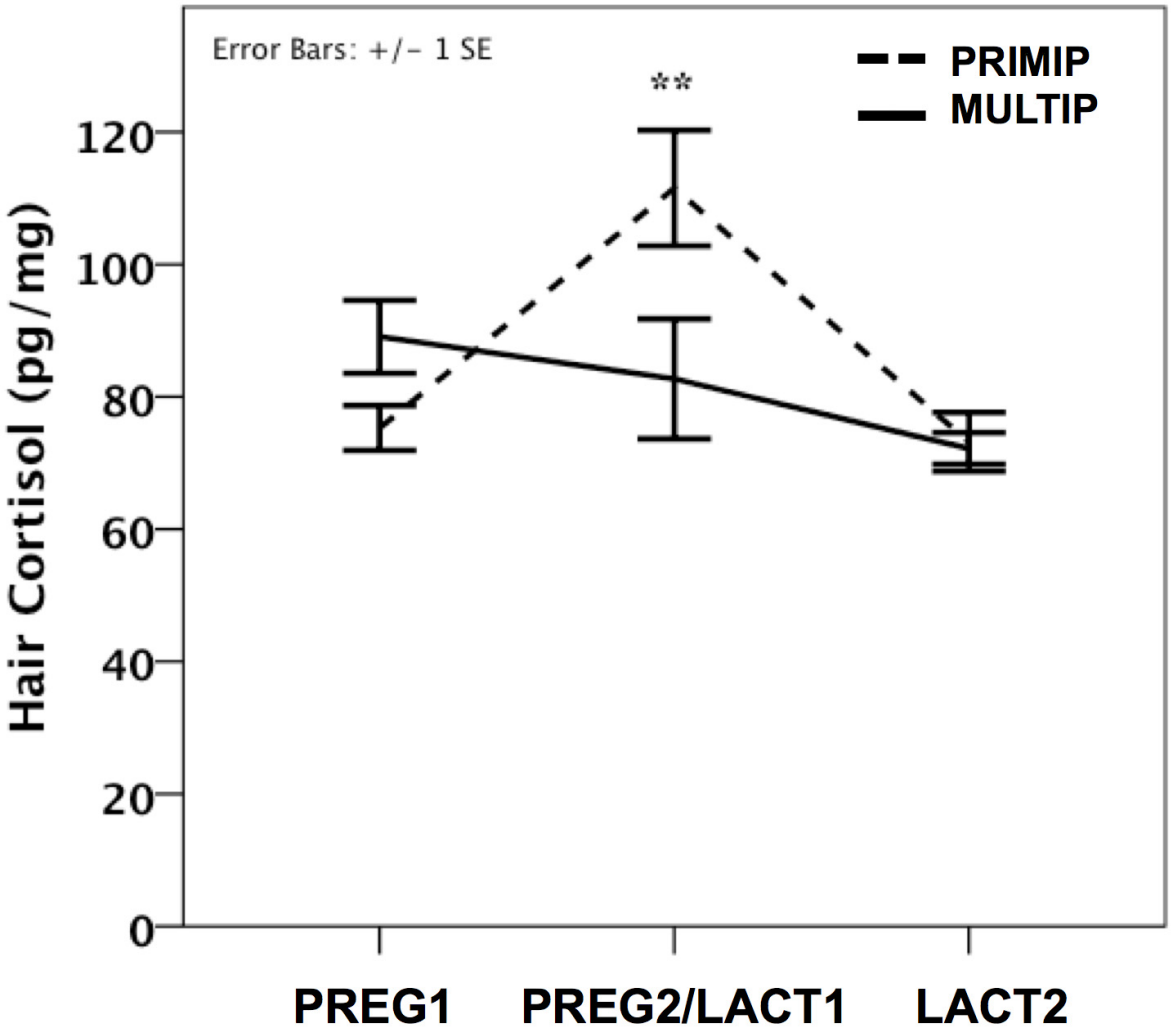
Parity differences in hair cortisol concentrations across pregnancy and lactation. PRIMP = primiparous, MULTIP = multiparous. PREG1 = mean±SE gestational age 124.9±8.7 days; PREG2/LACT1 = mean±SE gestational age 230.1±8.7 days or mean±SE postnatal age 60.1±8.7 days; LACT2 = mean±SE postnatal age 159.2±8.8 days. **p<0.01.

PRIMIP monkeys showed an elevation in HCCs during the PREG2/LACT1 period that was lacking in MULTIP monkeys (Fig 1). This differential pattern was borne out in the ANOVAs and post-hoc tests that revealed a main effect of parity (PRIMIPs > MULTIPs; F_(1)_ = 8.06, p = 0.01), a main effect of sampling period (F_(2)_ = 4.26, p = 0.02; PREG2/LACT1 > PREG1,p<0.05), and most importantly a significant parity x time interaction (F_(2)_ = 10.62, p<0.001).

### Hair progesterone concentrations (HPC)

**Fig 2:**
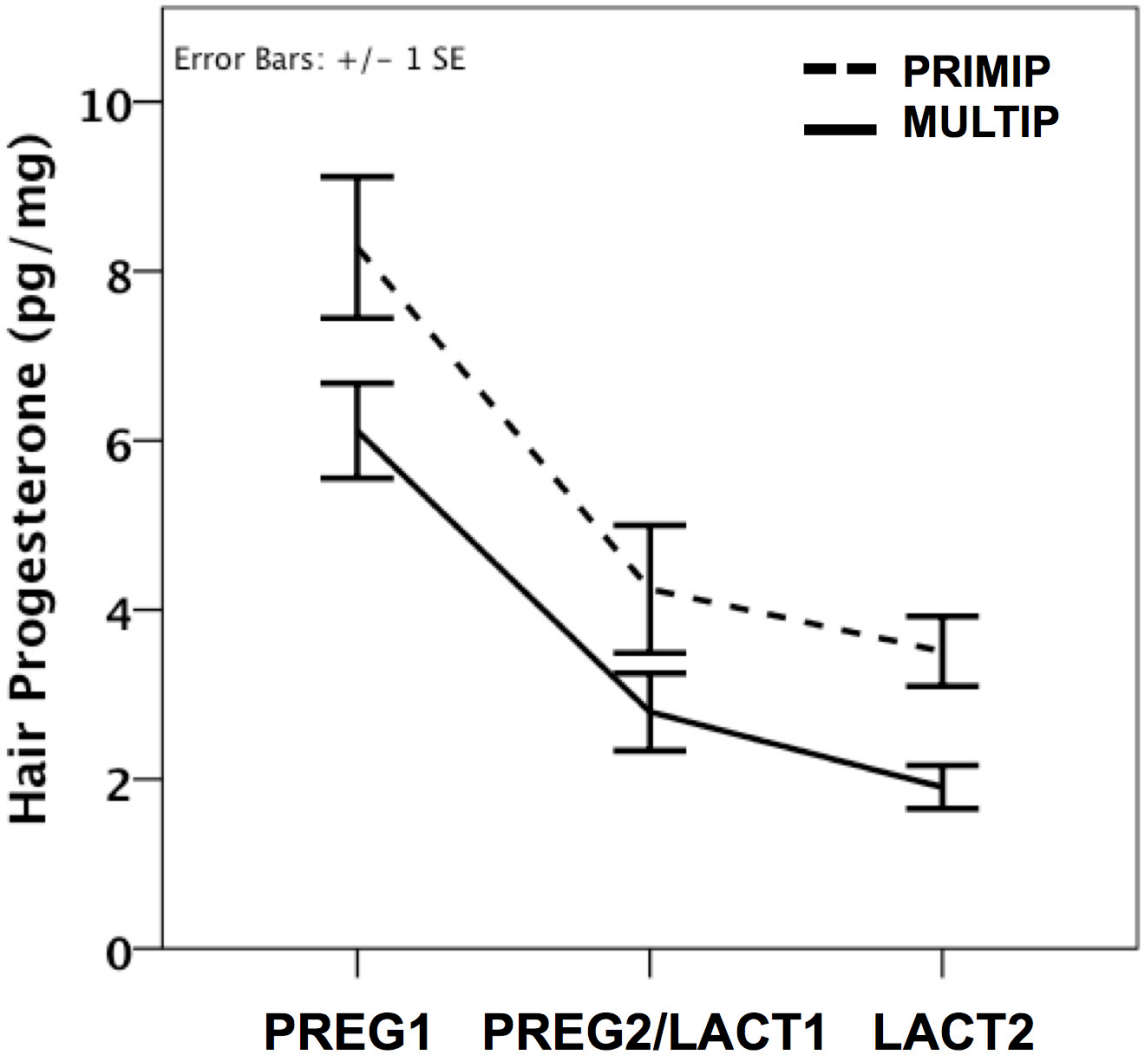
Parity differences in hair progesterone concentrations across pregnancy and lactation. PRIMP = primiparous, MULTIP = multiparous. PREG1 = mean±SE gestational age 124.9±8.7 days; PREG2/LACT1 = mean±SE gestational age 230.1±8.7 days or mean±SE postnatal age 60.1±8.7 days; LACT2 = mean±SE postnatal age 159.2±8.8 days. *p<0.05.

A significant effect of time was revealed (F_(2)_ = 7.16, p = 0.002), such that HPCs were highest in PREG1, then dropped significantly in PREG2/LACT1 (p<0.001) and declined again in LACT2 (p = 0.01; Fig 2). A main effect of parity was also observed (F_(1)_ = 6.06, p = 0.02), with PRIMIPs having higher overall HPCs than MULTIPS. No significant time x parity interaction was evident.

### Parity and neurobehavioral development

**Fig 3:**
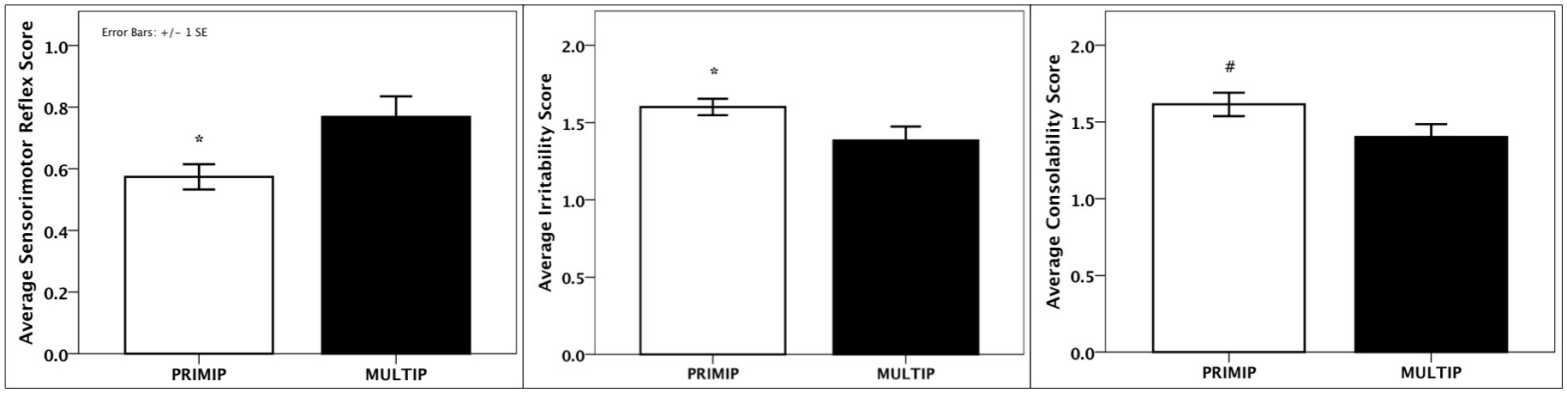
Parity differences in infant neurobehavioral development. PRIMP = primiparous, MULTIP = multiparous; *p<0.05; #p<0.10.

Infants of primiparous mothers had lower scores for sensorimotor reflexes (t_(24)_ = -2.48, p = 0.02), indicating poorer ability to orient to and follow visual and auditory stimuli. They also had higher scores of irritability (t_(24)_ = 2.08, p = 0.05) and tended to be more difficult to console (t_(24)_ = 1.85, p = 0.08; Fig 3).

### Hair hormone concentrations and neurobehavioral development

**Fig 4:**
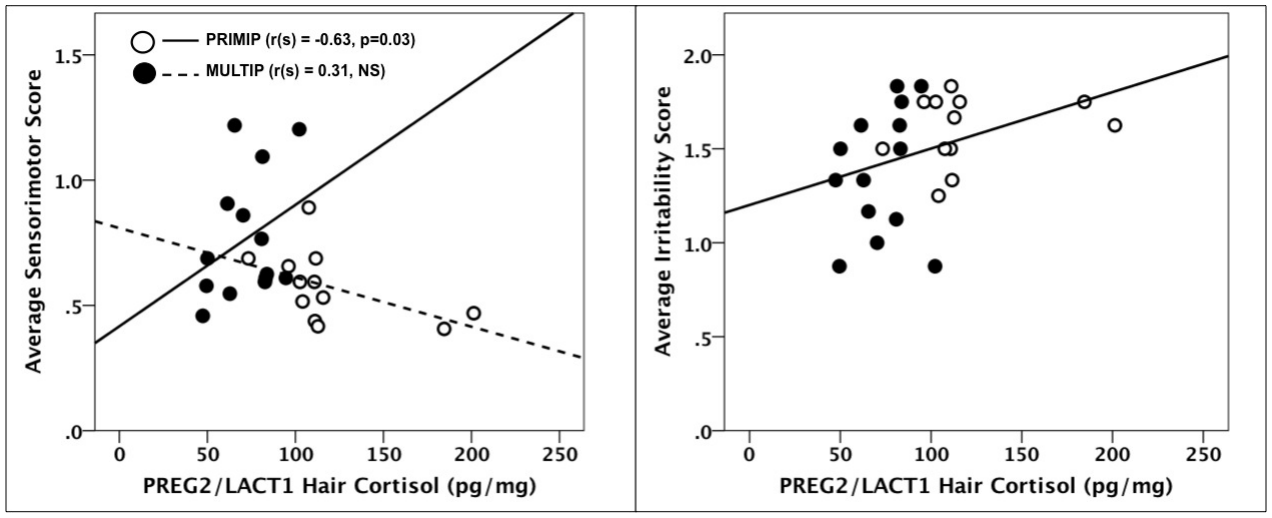
Associations between hair cortisol concentrations in late pregnancy/early lactation and infant neurobehavioral development. PRIMP = primiparous, MULTIP = multiparous.

Across all subjects, HCCs in PREG2/LACT1 were significantly negatively correlated with sensorimotor reflex scores (r_(s)_ = -0.41, p = 0.04) and positively with irritability scores (r_(s)_ = 0.43, p = 0.03). Further examination revealed that the correlation between HCCs and sensorimotor scores was significant for PRIMIPs only (r_(s)_ = -0.63, p = 0.03; Fig 4).

**Fig 5:**
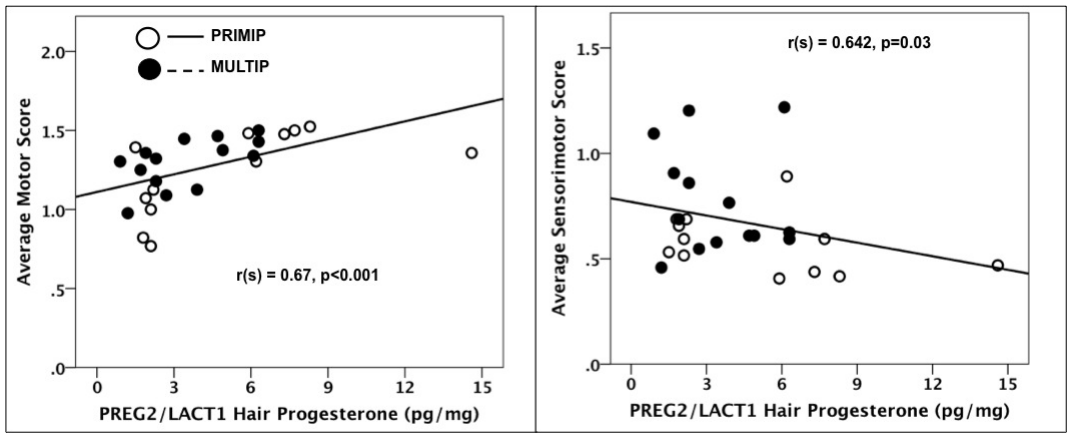
Associations between hair progesterone concentrations in late pregnancy/early lactation and infant neurobehavioral development. PRIMP = primiparous, MULTIP = multiparous.

HPCs in PREG2/LACT1 were significantly positively correlated with motor reflex scores (r_(s)_ = 0.67, p<0.001) and with sensorimotor reflex scores (r_(s)_ = 0.42, p = 0.03; Fig 5). No specific effects of parity for these correlations were revealed.

## Discussion

We determined, for the first time, hair cortisol and progesterone concentrations (as biomarkers of chronic levels of these hormones) across mid-to-late pregnancy and the period of lactation in rhesus monkeys, and we further found an effect of parity on these measures. Overall, we demonstrated that primiparous monkeys exhibit higher concentrations of both hair cortisol and hair progesterone, indicating that first pregnancies in macaque monkeys may result in noticeably greater hormonal changes than subsequent pregnancies. We also found infants born to first-time versus experienced mothers exhibited some differences in neurobehavioral development, and that chronic levels of maternal hormones were correlated with these outcomes.

We observed higher HCCs in our monkeys collectively during late pregnancy and early lactation than later in the postpartum period, which is in agreement with previous studies in humans and pigs [44, 47]. Primiparous monkeys exhibited significantly higher HCCs than multiparous monkeys at the end of pregnancy and in the first few months postpartum (Fig 1), findings which disagree with those of Kapoor and colleagues [41] showing no effect of parity on HCCs in rhesus monkeys three days after parturition. One major difference between the study of Kapoor et al. [41] and ours is that in theirs, mothers were singly caged with visual and auditory access to other monkeys whereas our monkeys were socially housed with 8–10 other adult females and an adult male. Postpartum mothers in Kapoor et al. [41] had average HCCs of 130.4+46.53 pg/mg (mean+SD), whereas in our study mothers in the neonatal period (PREG2/LACT1) had average HCCs of 94.12+36.03 (mean+SD), with PRIMIP mothers having average HCCs of 119.3+36.34 pg/mg and MULTIP mothers having HCCs of 72.54+17.08 pg/mg. We have previously reported significant differences in HCCs across different primate housing facilities for reasons that are not yet fully understood [60] In the present instance, it is possible that single housing resulted in higher overall HCCs for pregnant monkeys and that also masked differences between primiparous and experienced mothers. Alternatively, the rigors of the social hierarchy may have exerted a greater impact on our primiparous monkeys, thereby resulting in higher HCCs. However, Kapoor et al. [41] did find that primiparous mothers had higher hair concentrations of cortisone, an inactive cortisol metabolite, at birth. A significant portion of cortisol is converted to cortisone by the placenta, as well as by the hair shaft [41, 42]. Though we did not measure hair cortisone, it is possible that housing environment influences the cortisol:cortisone ratio in the hair of rhesus monkeys.

Numerous studies have demonstrated that primiparous monkeys are more anxious and protective of their infants than multiparous monkeys [61–63], and that they also receive more aggression from other adult females [57]. These collective physiological and environmental stressors, combined with the fact that primiparous monkeys are younger and are still developing during lactation when they are also nourishing their young, appear to result in more chronic activation of the HPA axis as evidenced by the elevated HCCs late in gestation and in the early postpartum period. Our findings have important implications for understanding HPA axis function in human pregnancy and lactation, as primiparous women have also demonstrated greater adjustment difficulties and higher stress in the days and weeks following birth compared to multiparous women [64, 65]. Moreover, neonatal exposure to maternal stress is known to impact infant development adversely [66–69], in part through ingestion of maternal glucocorticoids in mother’s milk [70–74]. Thus, studying chronic HPA axis activity during pregnancy and lactation via hair cortisol may be a useful tool for identifying mother-infant dyads at a higher risk for some of these negative consequences, such as delayed cognitive development and susceptibility to emotional health problems, particularly in young, first-time mothers.

We presented the first data on hair progesterone in a nonhuman primate species at multiple time points during pregnancy and lactation. As expected based on well-known patterns of plasma progesterone concentrations, HPCs were highest in pregnancy, lower during peak lactation, and at their lowest values later in lactation. Moreover, our HPC values across the study fall within the range of the values obtained by Kapoor et al. [41] in rhesus monkeys at birth using a different analytical method. Lactation is known to suppress ovarian steroid function in several mammalian species, including macaques [75]. Our findings of reduced progesterone concentrations in hair during lactation are in line with previously observed suppressed serum and fecal levels [2, 76, 77], and likely reflect the long-term suppression that occurs during this postpartum period. The present results along with those of Kapoor et al. [41] illustrate the usefulness of measuring hair progesterone as a biomarker of long-term levels of this hormone, similar to the growing use of hair cortisol as an index of chronic adrenocortical activity.

The higher overall HPCs in primiparous vs. multiparous monkeys may stem from the natural age-related decline in ovarian steroid production in older animals [78, 79]. Another possibility is that primiparous and multiparous monkeys differ in their production of placental progesterone, and/or in the amount of progesterone being utilized by the fetus for corticosteroid production [80, 81]. Additionally, perhaps the increased HPCs in primiparous monkeys are present to prime the induction of maternal behavior in these naïve females, as progesterone has been shown to induce such behaviors in nulliparous rodents [82, 83]. Further studies are necessary to address these hypotheses.

The major difference in hair progesterone between primiparous and multiparous monkeys occurred at the LACT2 sampling period (Fig 2). Given that the fall in progesterone after delivery of the infant triggers milk production [84], and that primiparous monkeys are known to produce less milk than multiparous monkeys [56], the higher HPCs in the primiparous animals may be related to this differential milk production while simultaneously revealing the increased allocation demands that first-time macaque mothers face since they must not only provide resources to their infants but also to their own growing bodies [56, 85, 86]

To our knowledge, the present findings are the first to demonstrate parity effects on infant neurobehavioral development in nonhuman primates. Given the differences in fetal/infant physical growth between primiparous and multiparous mothers [16–18, 87], it is not surprising that neurological differences would also be present. We also related offspring development to maternal hair hormone concentrations, and discovered significant correlations between maternal hair hormones in late pregnancy/early lactation and infant neurobehavioral measures. In particular, higher HCCs were associated with poorer sensorimotor reflex scores and with greater irritability, as predicted. These results are in agreement with previous studies linking greater maternal stress and higher short-term concentrations of cortisol (i.e., plasma, saliva) in pregnancy and the neonatal period with infant temperament [21, 22, 88]. They also align with preliminary results from Grant et al. [46] showing that the rise in maternal cortisol over pregnancy is correlated with fetal hair cortisol concentrations and delayed infant cognitive development. Thus, it is likely that chronic maternal circulating glucocorticoids, as measured by HCCs, program infant physiological, neurological and behavioral development. Moreover, this relationship is not only evident when mothers are stressed but (as shown in the present study) even in the absence of an applied stressor, when cortisol levels vary within the non-stress range. The extent of offspring exposure to maternal glucocorticoids via placental transfer [89] or through milk ingestion [73, 74] is not yet fully elucidated, nor is it clear whether such exposure is more important prenatally or during the early neonatal period in terms of programming subsequent offspring neurobehavioral development.

Our findings of higher maternal HPCs correlating with better infant motor reflex and sensorimotor scores are also the first to be documented in any species. The results are in line with studies of very pre-term human infants demonstrating that postnatal treatment with estradiol and progesterone results in normal psychomotor development and lung maturation [90, 91], and underscores the role of reproductive steroids on the developing nervous system. Similar to cortisol, it is possible that progesterone is being transferred via the placenta and/or mother’s milk (or some other mechanism), and future studies are needed to determine which mechanism or mechanisms are involved. However, these findings should be evaluated with caution, as our study was a correlational one and causal mechanisms between maternal hair hormones and infant development could not be determined.

The present study has several limitations that should be noted. The first, as already stated, is the correlational design of the study. For this reason, we cannot definitively ascribe differences in offspring development to variation in maternal hair hormone levels. Second, animals cannot be "randomly" assigned to parity status, as this is an outcome of their prior breeding history. Thus, we cannot rule out a possible influence of colony events that differed in the first pregnancies of the two sets of subjects. Additionally, the design of the study could be strengthened by collecting hair samples from pregnant monkeys at the same time throughout pregnancy rather than on the same calendar day. Unfortunately, the social housing environment precluded the repeated disturbances to the group that would have to occur in order for different monkeys to be captured on different days for hair sampling. Finally, we note that the hormones being measured in the mother’s hair may not originate exclusively from her tissues. An unknown portion of these hormones may originate from the fetus or the placenta [89, 92, 93], in which case maternal hair steroid levels are reflecting some output of fetal-placental communication. Further study is warranted to tease apart the sources of maternal hormones during the peripartum.

## Conclusions

Overall, the present findings show that primiparity is associated with a unique chronic hormonal profile across pregnancy and lactation, and in differential infant neurobehavioral development. Compared to multiparous animals, primiparous primates may experience greater physiological stress during the neonatal period, as evidenced by higher hair cortisol values, due to the combined demands of their own physical growth and their infant’s survival and sustenance. Primiparous monkeys also appear to produce more progesterone across pregnancy and early lactation, though the mechanisms underlying this observation require further investigation. Future studies will be able to utilize hormones in hair to determine physiological changes that mothers undergo as they transition from primiparity to multiparity. Moreover, studying hormones in hair will be useful for determining the long-term hormonal changes that pubertal/adolescent monkeys undergo as they transition from nulliparity to primiparity, and as such will serve as a valuable model for risky pregnancies in humans.

## Supporting Information

S1 Dataset Dataset for the current study in SPSS format. (SAV)

S2 Dataset Dataset for the current study in Excel format. (XLSX)
